# Long-term outcomes of catheter ablation for atrial fibrillation in octogenarians

**DOI:** 10.1007/s10840-024-01879-8

**Published:** 2024-08-14

**Authors:** Nikola Kozhuharov, Nabeela Karim, Antonio Creta, Lisa W. M. Leung, Rick Veasey, Armin Osmanagic, Anna Kefala, Mike Pope, Apostolos Vouliotis, Sven Knecht, Philipp Krisai, Pierre Jaïs, Claire Martin, Christian Sticherling, Matthew Ginks, Waqas Ullah, Richard Balasubramaniam, Manish Kalla, Mark M. Gallagher, Ross J. Hunter, Tom Wong, Dhiraj Gupta

**Affiliations:** 1 https://ror.org/01je02926 grid.437500.5 0000 0004 0489 5016 Liverpool Heart and Chest Hospital NHS Foundation Trust, Liverpool, UK; 2 https://ror.org/01q9sj412 grid.411656.1 0000 0004 0479 0855 Inselspital, University Hospital Bern, Bern, Switzerland; 3 https://ror.org/00cv4n034 grid.439338.6 0000 0001 1114 4366 Royal Brompton Hospital, London, UK; 4 https://ror.org/04fwa4t58 grid.413676.1 0000 0000 8683 5797 Harefield Hospital, London, UK; 5 https://ror.org/03g9ft432 grid.501049.9 The Barts Heart Centre, London, UK; 6 https://ror.org/0001ke483 grid.464688.0 0000 0001 2300 7844 . George’s Hospital, London, UK; 7 https://ror.org/01pjjvq50 grid.413704.5 0000 0004 0399 9710 Eastbourne District General Hospital, Eastbourne, UK; 8 https://ror.org/048emj907 grid.415490.d 0000 0001 2177 007X Queen Elizabeth Hospital Birmingham, Birmingham, UK; 9 https://ror.org/02pa0cy79 University Hospitals Dorset, Bournemouth, UK; 10 https://ror.org/03h2bh287 grid.410556.3 0000 0001 0440 1440 Oxford University Hospitals, Oxford, UK; 11 https://ror.org/0485axj58 grid.430506.4 University Hospitals Southampton, Southampton, UK; 12 https://ror.org/04k51q396 grid.410567.1 0000 0001 1882 505X University Hospital Basel, Basel, Switzerland; 13 https://ror.org/057qpr032 grid.412041.2 0000 0001 2106 639X Bordeaux University Hospital, Bordeaux, France; 14 https://ror.org/05mqgrb58 grid.417155.3 0000 0004 0399 2308 Royal Papworth Hospital, Cambridge, UK

**Keywords:** Atrial fibrillation ablation, Prognosis, Octogenarians

## Abstract

**Background and aims:**

Catheter ablation is superior to pharmacological therapy in controlling atrial fibrillation (AF). There are few data on the long-term outcome of AF ablation in octogenarian patients. This analysis aims to evaluate the outcome of AF ablation in octogenarians vs. younger patients.

**Methods:**

In this retrospective study in 13 centres in the UK, France, and Switzerland, the long-term outcomes of 473 consecutive octogenarian patients undergoing ablation for AF were compared to 473 matched younger controls (median age 81.3 [80.0, 83.0] vs. 64.4 [56.5, 70.7] years, 54.3% vs. 35.1% females; *p-*value for both < 0.001). The primary endpoint was the recurrence of atrial arrhythmia after a blanking period of 90 days within 365 days of follow-up.

**Results:**

Acute ablation success as defined as isolation of all pulmonary veins was achieved in 97% of octogenarians. Octogenarians experienced more procedural complications (11.4% vs 7.0%, *p* = 0.018). The median follow-up time was 281 [106, 365] days vs. 354 [220, 365] days for octogenarians vs. non-octogenarians (*p* < 0.001). Among octogenarians, 27.7% (131 patients) experienced a recurrence of atrial arrhythmia, in contrast to 23.5% (111 patients) in the younger group (odds ratio 1.49; 95% confidence interval 1.16–1.92; *p* = 0.002). In a multivariable regression model including gender, previous AF ablation, vascular disease, chronic kidney disease, CHA2DS2-VASc score, left atrial dilatation, and indwelling cardiac implantable electronic device, age above 80 remained an independent predictor of recurrence of arrhythmia.

**Conclusion:**

Ablation for AF is effective in octogenarians, but is associated with slightly higher procedural complication rate and recurrence of atrial arrhythmia than in younger patients.

**Graphical Abstract:**

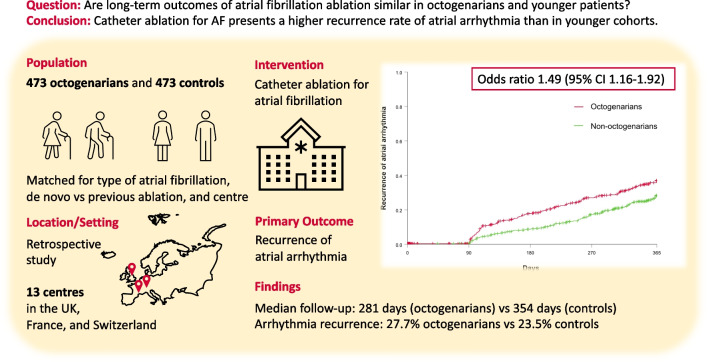

**Supplementary Information:**

The online version contains supplementary material available at 10.1007/s10840-024-01879-8.

## Introduction

Atrial fibrillation (AF) is the most common sustained arrhythmia, and is associated with increased morbidity and mortality [1, 2]. Catheter ablation, with pulmonary vein isolation (PVI) as its cornerstone, has proven to be an efficacious therapeutic strategy for AF, augmenting the quality of life and functional capacity of patients. [3–5]

The ageing population serves as a major risk factor for the incidence and prevalence of AF [6]. Projections suggest a more than twofold increase in the prevalence of AF by 2060, a surge attributable, at least in part, to the increase in life expectancy. Increasingly, octogenarians represent a significant proportion of patients referred for AF management. [2, 7, 8]

In octogenarian patients, the impact of AF on morbidity and mortality is even more accentuated. Medical management is particularly challenging due to the increased restrictions of the use of antiarrhythmic drugs in this patient group [2, 3]. Despite these realities, there is a paucity of data regarding catheter ablation’s acute and long-term outcomes in patients aged 80 and above. This data deficit is primarily due to the limited number of procedures in this age group and their systematic exclusion from most clinical trials [1, 3, 9–11]. Although a few relatively small studies have suggested that AF ablation in octogenarians may be safe and yield favourable success rates, [9, 11–14] concerns regarding potential procedural complications and limited efficacy continue to restrict the application of catheter ablation therapy in this demographic [15]. We hypothesised that contemporary atrial fibrillation ablation is as effective in octogenarians as in younger patients and sought to assess this hypothesis in a large, multicentre, international study.

## Methods

### Study population and design

In this retrospective, multicentre study, data from consecutive octogenarian and non-octogenarian patients undergoing AF ablation were collected from 11 tertiary hospitals in the UK (Supplemental Table 1), and 1 each in Switzerland (as part of the Swiss Atrial Fibrillation Pulmonary Vein Isolation Registry, ClinicalTrials.gov registry, Number NCT03718364) and France. The outcomes of octogenarian patients were compared to a matched cohort of non-octogenarian patients who underwent AF ablation within the same timeframe. The patients were matched on the following parameters: paroxysmal vs. non-paroxysmal AF, de novo vs. previous AF ablation, and the hospital of treatment. We excluded patients who had chosen to opt out of the use of their routinely collected clinical data for research purposes.

The study was carried out according to the principles of the Declaration of Helsinki and received approval from the relevant ethics committees. All patients granted their written informed consent for using their routinely collected clinical data in clinical research. The authors designed the study, gathered and analysed the data according to the STROBE guidelines for cohort studies (Supplemental Table 2) [16], and vouch for the data integrity and analysis.

### Ablation procedures

Catheter ablation included PVI and was performed either using point-by-point radiofrequency or with a cryoballoon ablation. All radiofrequency PVI procedures were performed with wide-area circumferential ablation using electroanatomical mapping and an irrigated tip contact force-sensing ablation catheter (CARTO 3, Biosense Webster Inc. Diamond Bar, CA, USA or Rhythmia, Boston Scientific, Marlborough, MA, USA, or EnSite NavX/Velocity, St. Jude Medical, Minneapolis, MN, USA). Following wide-area circumferential ablation, PVI was confirmed by documenting at least entrance block [3]. Additional lesions at the operators’ discretion were performed. These included but were not limited to left atrial posterior wall isolation. Cryoballoon ablation was performed using the 28-mm Arctic Front Advance cryoballoon (Medtronic Inc, Minneapolis, USA) and Achieve Advance Mapping catheter (Medtronic Inc, Minneapolis, USA). Cryoballoon ablation duration per vein was at least 180-s cryo-lesion aiming to achieve PVI and/or − 40 °C at 60 s, as previously described [17]. Additional cryo-applications were used according to the physician’s discretion.

### Patient follow-up and arrhythmia recurrence

Follow-up was performed according to clinical routine procedures at each participating centre, with the follow-up period for the primary outcome set at 365 days. According to the practice of the participating centres, patients were followed up in clinic visits every 3–4 months with an assessment of symptoms. The 12-lead ECG was performed at these visits; where appropriate, patients also received supplementary ambulatory ECG monitoring. Recurrence of atrial arrhythmia after an initial blanking period of 90 days was defined as at least one of the following: (1) need for further ablation for AF/atrial tachycardia (including cavotricuspid isthmus ablation); (2) need for DC cardioversion; (3) AF/atrial flutter or atrial tachycardia > 30 s recorded on ECG/Holter, or (4) in the opinion of the treating physician, experienced symptoms consistent with paroxysmal AF, even in the absence of documented AF/AT [2, 3]. Sensitivity analyses were conducted to verify the consistency of results, specifically excluding patients who had no ECG monitoring during follow-up, as well as those considered to have experienced a recurrence of atrial arrhythmia based solely on the physician’s assessment without any ECG confirmation.

### Outcome measures

The primary outcome measure was the recurrence of atrial arrhythmia after an initial blanking period of 90 days in octogenarian vs. non-octogenarian patients following atrial fibrillation ablation. Predefined subgroup analyses were performed to verify the consistency of the treatment effect. Secondary outcome measures encompassed comparisons of baseline and procedural characteristics between the octogenarian and non-octogenarian cohorts and predictors of the long-term success of AF ablation in octogenarians.

### Statistical analysis

The Kolmogorov–Smirnov test and visual inspection of the shape of the distribution of the variables were used to assess their normality. Continuous variables are presented as medians (with interquartile range), and categorical variables are as numbers and percentages. Comparisons between groups were made using Fisher’s exact test, Mann–Whitney *U* test, or Kruskal–Wallis test, as appropriate. The recurrence of atrial arrhythmia during follow-up was analysed using survival analysis for cumulative event rates, including Kaplan–Meier estimates and Cox regression for calculating hazard ratios. Interaction tests were conducted between the age groups and the prespecified subgroup variables using bivariate Cox regression models with tests for interaction to evaluate the consistency of treatment effects. The predefined subgroup variables included female gender, previous PVI, chronic kidney disease, ischemic heart disease, valvular heart disease, congestive heart failure, history of cerebrovascular accident/transient ischemic attack, left atrial dilatation, and left ventricular systolic dysfunction. Cox regression analyses were applied to identify predictors of atrial arrhythmia recurrence in univariable and multivariable analyses. The available baseline characteristics variables were treated as potentially confounding variables and were entered into the univariable model. Variables that were significant in that model were added to a multivariable model. No imputation was performed for missing values. No adjustments for multiple comparisons were made. All hypothesis testing was 2-sided, and a *p*-value < 0.05 was considered significant. This was an exploratory analysis within a retrospective study, and the sample size of the overall cohort was not explicitly determined for this analysis. Statistical analyses were carried out using SPSS/PC Software Package (version 25.0) and R Statistical Software (version 3.5.1).

## Results

### Patient demographics and characteristics

A total of 473 octogenarian patients with a median age of 81.3 [80.0, 83.0] were enrolled between January 2013 and June 2021. Of these, 222 (46.9%) had paroxysmal atrial fibrillation, and 251 (53.1%) had persistent AF. One hundred twenty-one (25.7%) had previous AF ablation (Table 1 and Supplemental Fig. 1). Following matching for the type of AF, previous AF ablation, and treating hospital, 473 non-octogenarian patients who underwent ablation in the same hospitals in the same timeframe with a median age of 64.4 [56.5, 70.7] were included in the analysis. In the octogenarian group, 257 (54.3%) patients were women vs. 166 (35.1%) in the non-octogenarian group (*p* < 0.001). Octogenarian patients had more comorbidities as compared to non-octogenarian patients, including hypertension (263 (55.6%) vs. 208 (44.0%), *p* < 0.001), diabetes (44 (9.3%) vs. 64 (13.5%), *p* < 0.041), previous history of CVA/TIA (42 (8.9%) vs. 19 (4.0%), *p* < 0.002), and presence of valvular heart disease (86 (18.2%) vs. 56 (11.8%), *p* < 0.006). Radiofrequency ablation was more utilised than cryoballoon PVI in octogenarians (327 (77.1%) vs. 289 (61.9%), *p* < 0.001). An indwelling permanent pacemaker or an ICD was more common among octogenarians (50 (10.7%) vs. 9 (1.9%) patients, *p* < 0.001). This provided continuous rhythm monitoring with a pacemaker, ICD, or implantable loop recorder in a greater proportion of octogenarian patients as compared to non-octogenarians (75 (18.8) vs. 28 (6.2%), *p* < 0.001).

**Table 1:** Baseline clinical characteristics, procedural characteristics, and follow-up characteristics in octogenarians vs. non-octogenarians

	Octogenarians	Non-octogenarians	*p*
Patient characteristics	(*n* = 473)	(*n* = 473)	
Age, years, median [IQR]	81.3 [80.0, 83.0]	64.4 [56.5, 70.7]	< 0.001
Female gender, *n* (%)	257 (54.3)	166 (35.1)	< 0.001
BMI, kg/m^2^, median [IQR]	26.3 [24.1, 29.1]	28.9 [25.3, 32.4]	0.546
Atrial fibrillation type, *n* (%)			< 0.001
Paroxysmal	222 (46.9)	222 (46.9)	
Persistent	217 (45.9)	225 (47.6)	
Long-standing persistent	34 (7.2)	26 (5.5)	
Previous AF ablation, *n* (%)	121 (25.7)	121 (25.7)	1
CHA2DS2 VASc score, median [IQR]	3.0 [3.0, 4.0]	2.0 [1.0, 3.0]	< 0.001
Prior history, *n* (%)			
CHF	67 (14.2)	54 (11.4)	0.21
Hypertension	263 (55.6)	208 (44.0)	< 0.001
Diabetes mellitus	44 (9.3)	64 (13.5)	0.041
CVA/TIA	42 (8.9)	19 (4.0)	0.002
Valvular heart disease	86 (18.2)	56 (11.8)	0.006
Ischemic heart disease	86 (18.2)	50 (10.6)	0.001
Other vascular diseases	17 (3.6)	7 (1.5)	0.039
Peripheral embolism	6 (1.3)	13 (2.7)	0.105
Renal impairment	79 (16.7)	27 (5.8)	< 0.001
Indwelling CIED, *n* (%)			< 0.001
PPM	50 (10.7)	9 (1.9)	
ICD or CRT	17 (3.6)	17 (3.6)	
LVEF, *n* (%)			0.765
Normal	355 (80.5)	382 (82.2)	
Mildly impaired	35 (7.9)	39 (8.4)	
Moderately impaired	23 (5.2)	21 (4.5)	
Severely impaired	28 (6.3)	23 (4.9)	
Left atrial size, *n* (%)			0.449
Normal	148 (42.3)	156 (43.0)	
Mildly enlarged	115 (32.9)	118 (32.5)	
Moderately enlarged	61 (17.4)	52 (14.3)	
Severely enlarged	26 (7.4)	37 (10.2)	
Procedural characteristics			
RF ablation, *n* (%)	327 (77.1)	289 (61.9)	< 0.001
Cryoballoon, *n*(%)	146 (22.9)	184 (38.1)	
Pulmonary vein isolation acute success, *n* (%)	428 (96.8)	445 (99.3)	0.006
OAC, *n* (%)			< 0.001
Uninterrupted DOAC	214 (60.1)	228 (50.0)	
Uninterrupted VKA	118 (33.1)	76 (16.7)	
Interrupted OAC	24 (6.7)	152 (33.3)	
Additional left atrial ablation, *n* (%)			
Any additional LA lines	145 (30.8)	123 (29.0)	0.562
PWI	12 (3.7)	3 (0.8)	0.011
CFAE	64 (13.7)	52 (12.8)	0.708
Cavotricuspid isthmus line, *n* (%)	113 (24.2)	107 (26.5)	0.438
Procedure time, min, median [IQR]	129.5 [90.0, 180.0]	130.0 [90.0, 180.0]	0.868
Acute complication, *n* (%)	54 (11.4)	33 (7.0)	0.018
Follow-up characteristics			
AAD on 12-month follow-up, *n* (%)			0.151
Flecainide or propafenone	20 (5.6)	10 (2.5)	
Amiodarone or dronedarone	17 (4.8)	24 (6.0)	
Sotalol	9 (2.5)	9 (2.2)	
Maximum rhythm monitoring during follow-up, *n* (%)			< 0.001
CIED or ILR	75 (18.8)	28 (6.2)	
Holter or event recorder	133 (33.4)	205 (45.5)	
12 lead ECG	172 (43.2)	193 (42.8)	
Reported symptoms only	18 (4.5)	25 (5.5)	
Recurrence of atrial arrhythmia, *n* (%)			0.037
Atrial fibrillation	56 (12.9)	48 (11.2)	
Atrial tachycardia	35 (8.1)	18 (4.2)	
Recurrence documentation based only on symptoms, *n* (%)	5 (12.5)	3 (6.7)	0.358
Ablation for atrial arrhythmia on follow-up	71 (18.5)	105 (25.0)	0.026

*AAD* antiarrhythmic drugs, *BMI* body mass index, *CHF* congestive heart failure, *CI* confidence interval, *CIED* cardiovascular implantable electronic device, *CFAE* complex fractionated atrial electrogram, *CVA/TIA* cerebrovascular accident/transient ischemic attack, *DOAC* direct oral anticoagulant, *ILR* implantable loop recorder, *IQR* interquartile range, *LVEF* left ventricular ejection fraction, *PWI* posterior wall isolation, *VKA* vitamin K antagonist

**Fig. 1:**
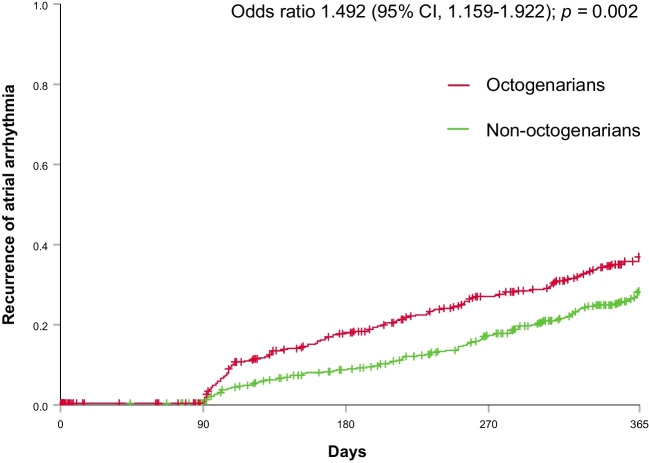
Recurrence of atrial arrhythmia throughout 12-month follow-up in octogenarian and non-octogenarian patients plotted in Kaplan–Meier curves. Recurrence of atrial arrhythmia after an initial blanking period of 90 days (for detailed definition, see *5. Definition of atrial arrhythmia recurrence during 12-month follow-up*); through day 365, the median follow-up time until reaching an event, completion of 365 follow-up, or patient lost to follow-up was 281 [106, 365] vs. 354 [220, 365] days for octogenarians vs. non-octogenarians (*p* < 0.001). CI, confidence interval

### Atrial fibrillation ablation complications

Acute complications were more common in the octogenarians’ group (54 (11.4%) vs. 33 (7.0%), *p* = 0.018). The most common adverse events following atrial fibrillation ablation were vascular access complications, in 18 (3.8%) octogenarians vs. non-octogenarians 13 (2.7%) and cardiac perforation and/or tamponade (8 (1.7%) vs 11 (2.3%), *p* = 0.013, Table 1).

### Recurrence of atrial arrhythmia

Among 946 patients in the overall cohort, the median follow-up time until reaching an event, completion of 365 days of follow-up, or patient lost to follow-up was 322 [153, 365] days for the overall cohort. The median follow-up time was significantly shorter for octogenarians at 281 [106, 365] days compared to non-octogenarians having a median follow-up of 354 [220, 365] days (*p* < 0.001). Over the 365-days follow-up period, 131 (28%) of the octogenarians had AF recurrence vs. 111 (23%) non-octogenarians. AF recurrence based purely on symptom assessment with no documentation on ECG was observed in 5 (12.5%) patients in the octogenarians group and in 3 (3.7%) patients in the non-octogenarian group (*p* = 0.466). Octogenarians were at increased risk of atrial arrhythmia recurrence (odds ratio 1.49, 95% CI 1.15–1.92; *p* = 0.002; Fig. 1). Predefined subgroup analyses showed consistent results in 9 of 9 subgroups (interaction *p*-value = ns for all predefined subgroups, including patients with paroxysmal vs. non-paroxysmal AF (Supplemental Table 3)).

**Fig. 2:**
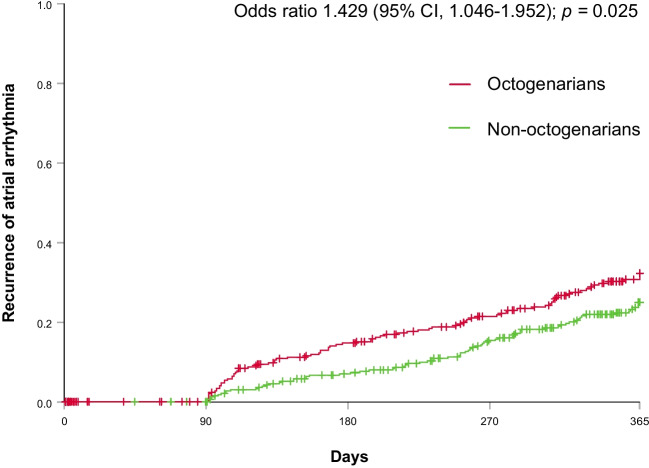
Recurrence of atrial arrhythmia throughout 12-month follow-up in octogenarian and non-octogenarian patients after de novo atrial fibrillation ablation plotted in Kaplan–Meier curves. Recurrence of atrial arrhythmia after an initial blanking period of 90 days (for detailed definition, see *5. Definition of atrial arrhythmia recurrence during 12-month follow-up*). CI, confidence interval

Previous AF ablation was more common in the group of octogenarian patients with recurrence of atrial arrhythmia (45 (34.4%) vs. 76 (22.4%), *p* = 0.008). After excluding patients with previous AF ablation, 86 (24.6%) of the octogenarians had AF recurrence vs. 73 (20.9%) non-octogenarians, odds ratio 1.429, 95% CI 1.046–1.952; *p* = 0.025 (Fig. 2).

Supplemental Table 4 illustrates the patients’ characteristics according to their recurrence of arrhythmia status for the 365-day follow-up. Patients who had a recurrence of arrhythmia within the 365-day follow-up were older (80.0 [66.0, 82.0] vs. 77.0 [63.4, 81.0] years, *p* = 0.031), had a higher proportion of females (123 (50.8%) vs. 300 (42.6%), *p* = 0.027), and more often had a history of previous atrial fibrillation ablation (81 (33.8%) vs. 161 (22.9%), *p* = 0.001). Patients’ characteristics of octogenarians with and without recurrence of atrial arrhythmia are presented in Supplemental Table 5.

All patients’ characteristics described in Table 1 were entered into univariable Cox proportional hazards models to predict the recurrence of atrial arrhythmia throughout 365 days of follow-up. Significant variables for the model were then entered into a multivariable Cox proportional hazards model (including age over 80). Tables 2 and 3 present the results for the overall cohort; Tables 4 and 5 present the results for the subgroup of octogenarians. In the multivariable model for the overall cohort, among other variables, age over 80 was an independent predictor for atrial arrhythmia recurrence (odds ratio 1.47 (95% CI 1.01–2.15), *p* = 0.044). In the multivariable analysis in the subgroup of octogenarians, female gender and previous AF ablation were independent predictors for recurrence of atrial arrhythmia (odds ratio 1.44 (95% CI 1.02–2.04) *p* = 0.041 and odds ratio 1.88 (95% CI 1.31–2.69, *p* = 0.001, respectively). Type of AF did not emerge as an independent predictor of AF recurrence in the multivariable models.

**Table 2:** Predictors of atrial arrhythmia recurrence throughout 12-month follow-up in the overall cohort

	Multivariable analysis*	
Variables	Odds ratio (95% CI)	*p*
Octogenarian	1.473 (95% CI 1.011–2.147)	0.044
Female gender	1.543 (95% CI1.116–2.133)	0.009
Previous atrial fibrillation ablation	1.843 (95% CI 1.349–2.517)	< 0.001
Vascular disease**	2.194 (95% CI 1.109–4.339)	0.024
Left atrial dilatation	1.381 (95% CI 1.010–1.889)	0.043

*250 cases with missing values and not considered for the multivariable analysis

**Vascular disease other than coronary artery disease or CVA/TIA

*CI* confidence interval, *CVA/TIA* cerebrovascular accident/transient ischemic attack

**Table 3:** Predictors of atrial arrhythmia recurrence throughout 12-month follow-up in the overall cohort, including atrial fibrillation type on multivariable analyses

	Univariable analysis		Multivariable analysis*	
Variables	Odds ratio (95% CI)	*p*	Odds ratio (95% CI)	*p*
Previous atrial fibrillation ablation	1.673 (95% CI 1.280–2.186)	< 0.001	1.823 (95% CI 1.333–2.492)	< 0.001
Female gender	1.435 (95% CI 1.116–1.847)	0.005	1.597 (95% CI 1.147–2.224)	0.006
Other vascular disease**	2.461 (95% CI 1.377–4.400)	0.002	2.179 (95% CI 1.101–4.315)	0.025
Octogenarian	1.492 (95% CI 1.159–1.922)	0.002	1.470 (95% CI 1.009–2.142)	0.045
Left atrial dilatation	1.402 (95% CI 1.040–1.891)	0.027	1.348 (95% CI 0.982–1.851)	0.064
Chronic kidney disease	1.633 (95% CI 1.155–2.309)	0.006	1.266 (95% CI 0.816–1.965)	0.292
CIED at baseline—any CIED	1.605 (95% CI 1.112–2.318)	0.012	1.301 (95% CI 0.840–2.017)	0.239
Type of AF at baseline—PAF	0.900 (95% CI 0.698–1.159)	0.414	0.857 (95% CI 0.625–1.174)	0.337
CHAD2S2 VASC score	1.108 (95% CI 1.025–1.197)	0.010	0.950 (95% CI 0.835–1.081)	0.438

*250 cases with missing values and not considered for the multivariable analysis

**Vascular disease other than coronary artery disease or CVA/TIA

*CI* confidence interval, *CIED* cardiovascular implantable electronic device, *CVA/TIA* cerebrovascular accident/transient ischemic attack, *PAF* paroxysmal atrial fibrillation

**Table 4:** Predictors of atrial arrhythmia recurrence throughout 12-month follow-up in octogenarians

	Multivariable analysis*	
Variables	Odds ratio (95% CI)	*p*
Female gender	1.438 (95% CI 1.015–2.038)	0.041
Previous atrial fibrillation ablation	1.877 (95% CI 1.307–2.694)	< 0.001

*2 cases with missing values and not considered for the multivariable analysis

*CI* confidence interval

**Table 5:** Univariable and multivariable predictors of atrial arrhythmia recurrence throughout 12-month follow-up, considering type of atrial fibrillation at baseline

	Univariable analysis		Multivariable analysis*	
Variables	Odds ratio (95% CI)	*p*	Odds ratio (95% CI)	*p*
Female gender	1.463 (95% CI 1.033–2.073)	0.032	1.434 (95% CI 1.007–2.042)	0.046
Previous atrial fibrillation ablation	1.883 (95% CI 1.312–2.703)	0.001	1.878 (95% CI 1.308–2.698)	< 0.001
Type of AF at baseline—PAF	1.053 (95% CI 0.747–1.484)	0.769	1.017 (95% CI 0.718–1.441)	0.922

*2 cases with missing values and not considered for the multivariable analysis

*CI* confidence interval; *PAF* paroxysmal atrial fibrillation

### Sensitivity analysis

When excluding cases where AF recurrence was assessed solely based on symptoms without ECG documentation, octogenarians demonstrated significantly higher recurrence rates, with an odds ratio of 1.45 (95% CI 1.11–1.88, *p* = 0.006). Similarly, when patients without any ECG follow-up were excluded, the recurrence rates among octogenarians were found to be higher, evidenced by an odds ratio of 1.44 (95% CI 1.11–1.88, *p* = 0.006).

## Discussion

This primary analysis within a large retrospective, international study aimed to evaluate the long-term outcomes of catheter ablation for atrial fibrillation in octogenarian vs. non-octogenarian patients. Among patients with paroxysmal and non-paroxysmal atrial fibrillation receiving a catheter-based therapy, age over 80 was associated with significantly higher atrial arrhythmia recurrence rates throughout 365 days of follow-up. Notably, the median follow-up duration was considerably shorter in the octogenarian group, which contributes to the less marked apparent difference in the raw event numbers observed.

The disparity in long-term success rates of catheter ablation among octogenarians can be attributed to several factors. This age group typically presents with a higher prevalence of comorbidities and established risk factors for atrial fibrillation, implying a greater likelihood of atrial scarring and a pro-arrhythmic substrate [18, 19]. Additionally, the presence of extra-pulmonary atrial fibrillation triggers have been shown to be more common in octogenarians [20, 21]. Notably, continuous rhythm monitoring with an indwelling permanent pacemaker, ICD, or ILR was more common among octogenarians, and one might expect this to explain the higher detection rates of atrial arrhythmia within this age group [22]. Nevertheless, in a multivariable model for predicting the recurrence of atrial arrhythmia demonstrated that age over 80 was an independent predictor of recurrence, while the presence of an indwelling permanent pacemaker, ICD, or ILR at the time of the procedure was not.

Additionally, the demographic analysis revealed a predominance of females in the octogenarian group. This gender difference may be explained by the generally higher life expectancy and relatively better health status of females, making them more likely to undergo interventions at older age.

The procedural complication rate was higher in octogenarians compared to younger patients. The main complications observed were related to vascular access, bleeding, and cardiac tamponade. These complications are more common in older adults due to increased frailty and comorbidities.

In our multivariable analysis, several factors such as age over 80 and previous AF ablation were independent predictors of AF recurrence. Although type of AF (paroxysmal vs. non-paroxysmal) was considered in our analyses, it did not independently predict AF recurrence, indicating that other factors may play a more significant role in recurrence risk among octogenarians.

This study substantiates and builds upon previous research regarding atrial fibrillation ablation in patients over 80. [9, 11–14, 23, 24] Previous trials, while informative, involved a relatively small number of octogenarian patients. These studies suggested comparable long-term outcomes regarding arrhythmia recurrence among octogenarians and non-octogenarians. However, due to the limited sample size in these studies, comparisons should be made with care, as neutral results could be partially attributed to a lack of statistical power. A prior meta-analysis suggested limited efficacy of AF ablation in patients over 75, but due to insufficient statistical power and data inconsistency within the octogenarian group, no specific conclusions could be drawn for this age group. [25]

Importantly, this study is the first of its kind, being a large, multicentre, international cohort exploring the outcomes of AF ablation in octogenarian vs. non-octogenarian patients. It not only examined a representative sample of both octogenarian and non-octogenarian patients but also considered recent advances in catheter ablation technology.

However, the study also bears some limitations. Primarily, the results are particular to octogenarians deemed suitable for PVI. The sample size also varied substantially across different participating centres, reflecting diverse eligibility criteria and thresholds for performing AF ablation in octogenarians across different hospitals. Further prospective studies are warranted to clarify the selection criteria for this age group. Despite efforts to minimise confounding factors through matching and multivariable regression analyses, some confounders may still exist. In a small subset of both octogenarians and non-octogenarians, arrhythmia recurrence was identified through typical symptoms reported and the treating physician’s judgement. While introducing a subjective element, the few recurrences diagnosed by this criterion did not affected the study’s overall findings as demonstrated in the sensitivity analyses that excluded these patients. One significant limitation of this study is the shorter median follow-up duration in the octogenarian group compared to the control group, with the follow-up period being approximately 20% shorter for octogenarians. This difference in follow-up duration is due to routine clinical follow-up procedures in observational settings, which could lead to shorter follow-up times in older patients who might have more frequent health issues or mobility constraints affecting their adherence to follow-up schedules. Although Cox regression and Kaplan–Meier analyses were used, employing censoring to deal with patients lost to follow-up, the shorter follow-up may still introduce bias by potentially underestimating the recurrence rates of atrial arrhythmia in this age group. To mitigate this bias, future prospective studies should strive to match follow-up durations more closely between age groups, ensuring more accurate comparisons. Despite this limitation, the higher observed recurrence rates in octogenarians are consistent with clinical expectations, suggesting that even with longer follow-up, the increased recurrence in older patients would likely persist. Additionally, potential selection bias should be considered. Octogenarians selected for catheter ablation in this study may have fewer comorbidities compared to the broader octogenarian population, which could limit the generalizability of our findings. Furthermore, while this manuscript provides an overview of procedural complications, a detailed examination of these complications is beyond the scope of the current analysis focused on long-term outcomes. A separate, in-depth analysis of the short-term outcomes and safety of AF catheter ablation in octogenarians is warranted to fully address the procedural risks associated with this intervention. This study predated the advent of Pulsed Field Ablation (PFA). Data was collected during a period of considerable advancements in cardiac ablation technology. Although technological diversity may have affected our findings, technological advancements should have similarly impacted the outcomes of both octogenarians and younger controls. Notably, beyond arrhythmia recurrence rates, the effect of AF on of cardiac function and quality of life in octogenarians must be addressed in future research. Importantly, while the primary outcome focuses on arrhythmia recurrence rates, the broader implications of AF ablation on cardiac function and quality of life in octogenarians warrant further investigation. Despite observing higher recurrence rates, AF ablation demonstrated favourable outcomes in this age group. Future research is needed to evaluate its impact on quality of life and patient-reported outcomes, particularly when comparing catheter ablation to the use of antiarrhythmic drugs, in octogenarians mostly limited to amiodarone, which is noted for its challenging safety and tolerance profile in this frail patient population.

In summary, our large international study comparing the outcomes of octogenarians vs. non-octogenarians undergoing catheter ablation for AF suggests that octogenarians experience higher recurrence rates of atrial arrhythmia and the potential causes behind this are multifactorial and need further study. Despite this, the long-term success rates for octogenarian patients are promising, affirming that AF ablation can be an effective treatment option for select individuals within this age group. Future research should focus on further refining patient selection criteria for this procedure in the octogenarian population, considering individual patient risk factors and the potential for improved quality of life and patient reported outcomes.

## Supplementary Information

Below is the link to the electronic supplementary material.

Supplementary file1 (DOCX 67.4 KB)

## Abbreviations

AF: Atrial fibrillation
CIED: Cardiovascular implantable electronic device
PVI: Pulmonary vein isolation
