# Supplementary material for: Long-term outcomes of catheter ablation for atrial fibrillation in octogenarians
Source: J Interv Card Electrophysiol. 2024 Aug 14;68(7):1513–22. doi: 10.1007/s10840-024-01879-8 (PMC12436546; doi:10.1007/s10840-024-01879-8)
Supplement: Supplementary file 1 — Supplementary file1 (DOCX 67.4 KB) [file 10840_2024_1879_MOESM1_ESM.docx]

# Supplemental Material

**Long-term outcomes of catheter ablation for atrial fibrillation in octogenarians**

**Supplemental Figure 1** Patient flow

473 octogenarians undergoing AF ablation in 11 UK, 1 Swiss, and 1 French sites

473 non-octogenarian controls included in this analysis

after matching for

study site, paroxysmal vs non-paroxysmal AF, and previous AF ablation

* Patients enrolled between January 2013 and June 2021. Median follow-up time until reaching an event, completion of 365 days’ follow-up, or patient lost to follow-up was 322 [153, 365] days. Among the patients without recurrence of arrhythmia, 140 patients had full 365 days’ follow-up.

AF = atrial fibrillation.

242 patients with recurrence

of atrial arrhythmia

131 octogenarians

704 patients without recurrence

of atrial arrhythmia

342 octogenarians

946 patients included

in this analysis*

**Supplemental Table 1** Number of patients recruited per study site.

| **Study site** | **Recruited patients, n (%)** |
| --- | --- |
| Barts Health NHS Trust, London, UK | 220 (23.3) |
| St George’s Hospital, London, UK | 208 (22.0) |
| NHB. Royal Brompton Hospital, London, UK | 104 (11.0) |
| Eastbourne District General Hospital, Eastbourne, UK | 74 (7.8) |
| HH. Harefield Hospital, London, UK | 70 (7.4) |
| Liverpool Heart and Chest Hospital NHS Trust, Liverpool, UK | 46 (4.9) |
| Queen Elizabeth Hospital Birmingham, Birmingham, UK | 40 (4.2) |
| University Hospitals Dorset, Bournemouth, UK | 40 (4.2) |
| Royal Papworth Hospital, Cambridge, UK | 38 (4.0) |
| University Hospitals Southampton, Southampton, UK | 38 (4.0) |
| Oxford University Hospitals, Oxford, UK | 26 (2.7) |
| University Hospital Basel, Basel, CH | 26 (2.7) |
| Bordeaux University Hospital, Bordeaux, FR | 16 (1.7) |

**Supplemental Table 2.**

|  | Item No | Recommendation | Page No |
| --- | --- | --- | --- |
| **Title and abstract** | 1 | (*a*) Indicate the study’s design with a commonly used term in the title or the abstract | 1 |
|  |  | (*b*) Provide in the abstract an informative and balanced summary of what was done and what was found | 3 |
| Introduction | | | |
| Background/rationale | 2 | Explain the scientific background and rationale for the investigation being reported | 4 |
| Objectives | 3 | State specific objectives, including any prespecified hypotheses | 4 |
| Methods | | | |
| Study design | 4 | Present key elements of study design early in the paper | 5 |
| Setting | 5 | Describe the setting, locations, and relevant dates, including periods of recruitment, exposure, follow-up, and data collection | 5 |
| Participants | 6 | (*a*) Give the eligibility criteria, and the sources and methods of selection of participants. Describe methods of follow-up | 5-6 |
|  |  | (*b*) For matched studies, give matching criteria and number of exposed and unexposed |  |
| Variables | 7 | Clearly define all outcomes, exposures, predictors, potential confounders, and effect modifiers. Give diagnostic criteria, if applicable | 6-7 |
| Data sources/ measurement | 8* | For each variable of interest, give sources of data and details of methods of assessment (measurement). Describe comparability of assessment methods if there is more than one group | 6-7 |
| Bias | 9 | Describe any efforts to address potential sources of bias | 7-8 |
| Study size | 10 | Explain how the study size was arrived at | n.a. |
| Quantitative variables | 11 | Explain how quantitative variables were handled in the analyses. If applicable, describe which groupings were chosen and why | 7 |
| Statistical methods | 12 | (*a*) Describe all statistical methods, including those used to control for confounding | 7-8 |
|  |  | (*b*) Describe any methods used to examine subgroups and interactions | 8-8 |
|  |  | (*c*) Explain how missing data were addressed | 7-8 |
|  |  | (*d*) If applicable, explain how loss to follow-up was addressed | 7-8 |
|  |  | (*e*) Describe any sensitivity analyses | 7-8 |
| Results | | |  |
| Participants | 13* | (a) Report numbers of individuals at each stage of study—eg numbers potentially eligible, examined for eligibility, confirmed eligible, included in the study, completing follow-up, and analysed | 9 |
|  |  | (b) Give reasons for non-participation at each stage | n.a. |
|  |  | (c) Consider use of a flow diagram | Figure S1 |
| Descriptive data | 14* | (a) Give characteristics of study participants (eg demographic, clinical, social) and information on exposures and potential confounders | 9 |
|  |  | (b) Indicate number of participants with missing data for each variable of interest | Table 2 |
|  |  | (c) Summarise follow-up time (eg, average and total amount) | 9-10 |
| Outcome data | 15* | Report numbers of outcome events or summary measures over time | 10 |
| Main results | 16 | (a) Give unadjusted estimates and, if applicable, confounder-adjusted estimates and their precision (eg, 95% confidence interval). Make clear which confounders were adjusted for and why they were included | 10 |
|  |  | (b) Report category boundaries when continuous variables were categorized | 9-10 |
|  |  | (c) If relevant, consider translating estimates of relative risk into absolute risk for a meaningful time period | - |
| Other analyses | 17 | Report other analyses done—eg analyses of subgroups and interactions, and sensitivity analyses | 11 |
| Discussion | | | |
| Key results | 18 | Summarise key results with reference to study objectives | 12-13 |
| Limitations | 19 | Discuss limitations of the study, taking into account sources of potential bias or imprecision. Discuss both direction and magnitude of any potential bias | 14 |
| Interpretation | 20 | Give a cautious overall interpretation of results considering objectives, limitations, multiplicity of analyses, results from similar studies, and other relevant evidence | 12-14 |
| Generalisability | 21 | Discuss the generalisability (external validity) of the study results | 12-14 |
| Other information | | | |
| Funding | 22 | Give the source of funding and the role of the funders for the present study and, if applicable, for the original study on which the present article is based | 15 |

*Give information separately for exposed and unexposed groups.

**Supplemental Table 3** Interaction p-values in bivariable models using a cox proportional hazard analysis for predicting atrial arrhythmia recurrence at 12 months including octogenarians age group and predefined subgroup variables.

| **Predefined subgroup variables** | ***p* of interaction** |
| --- | --- |
| Female gender | 0.495 |
| Previous atrial fibrillation ablation | 0.358 |
| Chronic kidney disease | 0.964 |
| Ischemic heart disease | 0.660 |
| CVA/TIA | 0.430 |
| Valvular heart disease | 0.394 |
| Congestive heart failure | 0.705 |
| Left atrial dilatation | 0.743 |
| Left ventricular systolic dysfunction | 0.255 |

CVA/TIA = cerebrovascular accident/transient ischemic attack**.**

**Supplemental Table 4** Baseline clinical characteristics, procedural characteristics and follow-up characteristics according to freedom from arrhythmia at 12 months’ follow-up in the overall cohort.

|  | **Recurrence of atrial arrhythmia** | **Freedom from atrial arrhythmia** | ***p*** |
| --- | --- | --- | --- |
| **Patient characteristics** | (n= 242, 26%) | (n= 704, 74%) |  |
| Age, years, median [IQR] | 80.0 [66.0, 82.0] | 77.0 [63.4, 81.0] | 0.031 |
| Female gender, n (%) | 123 (50.8) | 300 (42.6) | 0.027 |
| BMI, kg/m^2^, median [IQR] | 27.3 [24.8, 30.9] | 27.5 [24.6, 30.9] | 0.638 |
| Atrial fibrillation type, n (%) |  |  | 0.577 |
| Paroxysmal | 108 (44.6) | 336 (47.7) |  |
| Persistent | 120 (49.6) | 322 (45.7) |  |
| Long-standing persistent | 14 (5.8) | 46 (6.5) |  |
| Previous Afib ablation, n (%) | 81 (33.8) | 161 (22.9) | 0.001 |
| CHA2DS2 VASc score, median [IQR] | 3.0 [2.0, 4.0] | 3.0 [1.8, 4.0] | 0.096 |
| Prior history, n (%) | 3.0 [2.0, 4.0] |  | 0.096 |
| CHF | 25 (10.4) | 96 (13.6) | 0.191 |
| Hypertension | 122 (50.4) | 349 (49.6) | 0.822 |
| Diabetes mellitus | 26 (10.7) | 82 (11.6) | 0.703 |
| CVA/TIA | 14 (5.8) | 47 (6.7) | 0.626 |
| Valvular heart disease | 46 (19.0) | 96 (13.6) | 0.044 |
| Ischemic heart disease | 38 (15.7) | 98 (13.9) | 0.495 |
| Other vascular disease | 12 (5.0) | 12 (1.7) | 0.005 |
| Peripheral embolism | 5 (2.1) | 14 (2.0) | 0.941 |
| Renal impairment | 38 (15.8) | 68 (9.7) | 0.01 |
| Indwelling CIED, n (%) |  |  | 0.068 |
| PPM | 22 (9.1) | 37 (5.3) |  |
| ICD or CRT | 11 (4.5) | 23 (3.3) |  |
| LVEF, n (%) |  |  | 0.433 |
| Normal | 192 (83.5) | 545 (80.6) |  |
| Mildly impaired | 16 (7.0) | 58 (8.6) |  |
| Moderately impaired | 13 (5.7) | 31 (4.6) |  |
| Severely impaired | 9 (3.9) | 42 (6.2) |  |
| Left atrial size, n (%) |  |  | 0.04 |
| Normal | 68 (36.8) | 236 (44.7) |  |
| Mildly enlarged | 75 (40.5) | 158 (29.9) |  |
| Moderately enlarged | 30 (16.2) | 83 (15.7) |  |
| Severely enlarged | 12 (6.5) | 51 (9.7) |  |
| **Procedural characteristics** |  |  |  |
| RF ablation, n (%) | 156 (72.2) | 460 (68.1) | 0.259 |
| Pulmonary vein isolation acute success, n (%) | 217 (96.4) | 656 (98.6) | 0.037 |
| OAC, n (%) |  |  | 0.109 |
| Uninterrupted DOAC | 112 (54.1) | 330 (54.5) |  |
| Uninterrupted VKA | 41 (19.8) | 153 (25.3) |  |
| Interrupted OAC | 54 (26.1) | 122 (20.2) |  |
| Additional left atrial ablation, n (%) |  |  |  |
| Any additional LA lines | 79 (34.2) | 189 (28.5) | 0.101 |
| PWI | 2 (1.1) | 13 (2.6) | 0.259 |
| CFAE | 37 (16.8) | 79 (12.1) | 0.076 |
| Cavotricuspid isthmus line, n (%) | 54 (24.5) | 166 (25.5) | 0.778 |
| Procedure time, min, median [IQR] | 138.0 [90.8, 180.0] | 126.0 [90.0, 180.0] | 0.686 |
| Acute complication, n (%) | 29 (12.0) | 58 (8.2) | 0.082 |
| Acute complication type, n (%) |  |  | 0.679 |
| Cardiac perforation and/or tamponade | 4 (13.8) | 15 (25.9) |  |
| Palsy of phrenic or vagal nerve | 4 (13.8) | 7 (12.1) |  |
| Vascular access complication | 10 (34.5) | 21 (36.2) |  |
| Significant brady or tachyarrhythmia | 3 (10.3) | 3 (5.2) |  |
| Oher - requiring prolongation of hospitalisation or early readmission | 6 (20.7) | 7 (12.1) |  |
| Other - not requiring prolongation of hospitalisation or early readmission | 2 (6.9) | 5 (8.6) |  |
| **Follow-up characteristics** |  |  |  |
| AAD on 12 months’ follow-up, n (%) |  |  | 0.231 |
| None | 169 (86.2) | 500 (89.0) |  |
| Flecainide or Propafenone | 12 (6.1) | 18 (3.2) |  |
| Amiodarone or Dronedarone | 12 (6.1) | 29 (5.2) |  |
| Sotalol | 3 (1.5) | 15 (2.7) |  |
| Maximum rhythm monitoring during follow-up, n (%) |  |  | 0.001 |
| Reported symptoms only | 9 (3.9) | 34 (5.5) |  |
| 12 lead ECG | 121 (52.4) | 244 (39.5) |  |
| Holter or event recorder | 69 (29.9) | 269 (43.5) |  |
| CIED or ILR | 32 (13.9) | 71 (11.5) |  |

AAD = antiarrhythmic drugs; BMI = body mass index; CHF = congestive heart failure; CI = confidence interval; CIED = cardiovascular implantable electronic device; CFAE = complex fractionated atrial electrogram; CVA/TIA = cerebrovascular accident/transient ischemic attack; DOAC = direct oral anticoagulant; ILR = implantable loop recorder; IQR = interquartile range; LVEF = left ventricular ejection fraction; PWI = posterior wall isolation; VKA = Vitamin K antagonist.

**Supplemental Table 5** Baseline clinical characteristics, procedural characteristics and follow-up characteristics according to freedom from arrhythmia at 12 months’ follow-up in octogenarians.

|  | **Recurrence of atrial arrhythmia** | **Freedom from atrial arrhythmia** | ***p*** |
| --- | --- | --- | --- |
| **Patient characteristics** | (n= 131, 28%) | (n= 342, 72%) |  |
| Age, years, median [IQR] | 81.6 [80.0, 83.0] | 81.1 [80.0, 83.0] | 0.559 |
| Female gender, n (%) | 77 (58.8) | 180 (52.6) | 0.23 |
| BMI, kg/m^2^, median [IQR] | 26.8 [23.6, 30.3] | 26.0 [24.2, 29.0] | 0.484 |
| Atrial fibrillation type, n (%) |  |  | 0.984 |
| Paroxysmal | 62 (47.3) | 160 (46.8) |  |
| Persistent | 60 (45.8) | 157 (45.9) |  |
| Long-standing persistent | 9 (6.9) | 25 (7.3) |  |
| Previous Afib ablation, n (%) | 45 (34.4) | 76 (22.4) | 0.008 |
| CHA2DS2 VASc score, median [IQR] | 4.0 [3.0, 4.0] | 3.0 [3.0, 4.0] | 0.625 |
| Prior history, n (%) | 4.0 [3.0, 4.0] | 3.0 [3.0, 4.0] | 0.625 |
| CHF | 13 (9.9) | 54 (15.8) | 0.102 |
| Hypertension | 69 (52.7) | 194 (56.7) | 0.427 |
| Diabetes mellitus | 15 (11.5) | 29 (8.5) | 0.32 |
| CVA/TIA | 9 (6.9) | 33 (9.6) | 0.342 |
| Valvular heart disease | 31 (23.7) | 55 (16.1) | 0.056 |
| Ischemic heart disease | 25 (19.1) | 61 (17.8) | 0.753 |
| Other vascular disease | 8 (6.1) | 9 (2.6) | 0.069 |
| Peripheral embolism | 2 (1.5) | 4 (1.2) | 0.756 |
| Renal impairment | 29 (22.1) | 50 (14.6) | 0.05 |
| Indwelling CIED, n (%) |  |  | 0.234 |
| PPM | 19 (14.5) | 31 (9.2) |  |
| ICD or CRT | 4 (3.1) | 13 (3.8) |  |
| LVEF, n (%) |  |  | 0.131 |
| Normal | 104 (86.0) | 251 (78.4) |  |
| Mildly impaired | 7 (5.8) | 28 (8.8) |  |
| Moderately impaired | 7 (5.8) | 16 (5.0) |  |
| Severely impaired | 3 (2.5) | 25 (7.8) |  |
| Left atrial size, n (%) |  |  | 0.075 |
| Normal | 37 (37.0) | 111 (44.4) |  |
| Mildly enlarged | 43 (43.0) | 72 (28.8) |  |
| Moderately enlarged | 15 (15.0) | 46 (18.4) |  |
| Severely enlarged | 5 (5.0) | 21 (8.4) |  |
| **Procedural characteristics** |  |  |  |
| RF ablation, n (%) | 87 (81.3) | 240 (75.7) | 0.233 |
| Pulmonary vein isolation acute success, n (%) | 114 (94.2) | 314 (97.8) | 0.054 |
| OAC, n (%) |  |  | 0.195 |
| Uninterrupted DOAC | 66 (66.0) | 148 (57.8) |  |
| Uninterrupted VKA | 26 (26.0) | 92 (35.9) |  |
| Interrupted OAC | 8 (8.0) | 16 (6.2) |  |
| Additional left atrial ablation, n (%) |  |  |  |
| Any additional LA lines | 44 (33.8) | 101 (29.6) | 0.374 |
| PWI | 2 (2.2) | 10 (4.4) | 0.349 |
| CFAE | 22 (17.3) | 42 (12.4) | 0.165 |
| Cavotricuspid isthmus line, n (%) | 33 (25.8) | 80 (23.6) | 0.623 |
| Procedure time, min, median [IQR] | 143.5 [93.8, 181.8] | 120.0 [90.0, 180.0] | 0.423 |
| Acute complication, n (%) | 19 (14.5) | 35 (10.2) | 0.191 |
| Acute complication type, n (%) |  |  | 0.612 |
| Cardiac perforation and/or tamponade | 2 (10.5) | 6 (17.1) |  |
| Palsy of phrenic or vagal nerve | 3 (15.8) | 6 (17.1) |  |
| Vascular access complication | 6 (31.6) | 12 (34.3) |  |
| Significant brady or tachyarrhythmia | 3 (15.8) | 3 (8.6) |  |
| Oher - requiring prolongation of hospitalisation or early readmission | 5 (26.3) | 5 (14.3) |  |
| Other - not requiring prolongation of hospitalisation or early readmission | 0 (0.0) | 3 (8.6) |  |
| **Follow-up characteristics** |  |  |  |
| Any AAD on 12 months’ follow-up, n (%) | 11 (10.9) | 35 (13.7) | 0.48 |
| AAD on 12 months’ follow-up, n (%) |  |  | 0.822 |
| None | 90 (89.1) | 221 (86.3) |  |
| Flecainide or Propafenone | 4 (4.0) | 16 (6.2) |  |
| Amiodarone or Dronedarone | 5 (5.0) | 12 (4.7) |  |
| Sotalol | 2 (2.0) | 7 (2.7) |  |
| Maximum rhythm monitoring during follow-up, n (%) |  |  | 0.043 |
| Reported symptoms only | 4 (3.3) | 14 (5.1) |  |
| 12 lead ECG | 65 (53.3) | 107 (38.8) |  |
| Holter or event recorder | 31 (25.4) | 102 (37.0) |  |
| CIED | 22 (18.0) | 53 (19.2) |  |

AAD = antiarrhythmic drugs; BMI = body mass index; CHF = congestive heart failure; CI = confidence interval; CIED = cardiovascular implantable electronic device; CFAE = complex fractionated atrial electrogram; CVA/TIA = cerebrovascular accident/transient ischemic attack; DOAC = direct oral anticoagulant; IQR = interquartile range; LVEF = left ventricular ejection fraction; PWI = posterior wall isolation; VKA = Vitamin K antagonist.
